# Exploring the Interplay Between Thyroid Hormone Levels and Symptoms of Anxiety and Depression in Anorexia Nervosa

**DOI:** 10.1002/brb3.70685

**Published:** 2025-07-11

**Authors:** Lama Mattar, Anne‐Laure Delaunay, Sylvie Berthoz, Maeva Duquesnoy, Sylvain Iceta, Christophe Lalane, EVHAN group, Mouna Hanachi, Nathalie Godart

**Affiliations:** ^1^ Nutrition Program, Department of Nutrition and Food Science, School of Arts and Sciences Lebanese American University Beirut Lebanon; ^2^ Service Universitaire De Psychiatrie De L'enfant Et De L'adolescent Centre Hospitalier de Versailles Versailles France; ^3^ Univ. Bordeaux, INCIA, CNRS, UMR 5287, F‐33000 Bordeaux France Bordeaux France; ^4^ Paris‐Saclay University, INRAE, AgroParisTech MICALIS Institute Jouy‐en‐Josas France; ^5^ Research Center of the Quebec Heart and Lung Institute Québec QC Canada; ^6^ Department of Psychiatry and Neurosciences Laval University Québec QC Canada; ^7^ Université Paris Cité Paris France; ^8^ Clinical Nutrition department, Paul Brousse University Hospital, Assistance Publique‐Hôpitaux de Paris (AP‐HP) Villejuif France; ^9^ Paris‐Saclay University, INRAE, AgroParisTech MICALIS institute Jouy‐en‐Josas France; ^10^ Paris‐Saclay University, Le Kremlin‐Bicêtre Gif‐sur‐Yvette France; ^11^ UFR Simone Veil, UVSQ Montigny le Bretonneux France; ^12^ Fondation Santé des Etudiants de France Paris France; ^13^ Équipe INSERM Psychiatrie du développement, Centre de Recherche En épidémiologie et Santé Des Populations, UMR 1018 Paris‐Saclay Université, Université Versailles‐Saint‐ Quentin‐En‐Yvelines Montigny‐Le‐Bretonneux France; ^14^ Department of Psychiatry for Adolescents and Young Adults, Institut Mutualiste Montsouris Paris France

**Keywords:** anorexia nervosa, anxiety, depression, malnutrition, obsessive‐compulsive, thyroid hormone levels

## Abstract

**Objective:**

A strong association exists between anorexia nervosa (AN) and symptoms of depression, anxiety, and obsessive‐compulsive disorder (OCD). The levels of these psychiatric symptoms observed in AN may be influenced by different biological factors related to poor nutritional status and changes in thyroid hormone levels. Yet, few studies have investigated this relationship. The objective of this study is to examine the association between depression, anxiety, and obsessive‐compulsive (OC) symptoms and circulating thyroid hormones in a sample of undernourished patients with AN.

**Methods:**

Two hundred and two patients with AN (DSM‐IV TR) were included in the study and were assessed upon admission for duration of illness, psychiatric treatments, sociodemographic data, and different psychopathological symptoms [depression (BDI), anxiety (HAD scale), obsessive‐compulsive (MOCI), social phobia (LSAS fear sub‐scale), and Eating Attitudes Test‐26 (EAT‐26)] using psychometric scales. Nutritional status was assessed with body mass index (BMI) and body composition using bioelectrical impedance. Upon patient admission, free‐T3 (fT3), free‐T4 (fT4), and TSH thyroid hormone plasma levels were collected, as well as albumin and transthyretin levels.

**Results:**

Taking into consideration confounding factors, particularly the duration of AN evolution, thyroid hormone (fT3 and/or fT4) blood levels can partially explain the levels of depression and OCD symptoms of the doubt type and social phobia in undernourished AN patients.

**Conclusion:**

A high prevalence of these symptoms among malnourished individuals requires investigation to differentiate between symptoms directly related to the biological effects of malnutrition and those indicative of a comorbid condition such as depression or anxiety.

## Graphical abstract

This study evaluates the association between depression, anxiety, and obsessive‐compulsive symptoms and circulating thyroid hormones in a sample of undernourished anorexia nervosa patients. Using the longitudinal research project, EVHAN, two hundred and two anorexia nervosa (AN) patients were recruited. We found that thyroid hormone levels may account in part for the levels of depression and obsessive‐compulsive symptoms in undernourished anorexia nervosa patients.

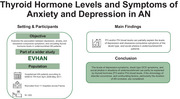

## 1 Introduction

Anorexia nervosa (AN) has one of the highest mortality rates among psychiatric illnesses (Berkman et al. 2007) as a result of the complications of undernourishment and suicide risk (Smink et al. 2012). AN is strongly associated with depression, anxiety, and obsessive‐compulsive (OC) symptoms (Godart et al. 2002; Mattar et al. 2011; Mattar et al. 2012; Mattar et al. 2012; Pleplé et al. 2021). Some of these symptoms are part of AN itself (weight loss, fear of eating in public, compulsive physical activity, etc.); others can be the direct consequences of undernourishment (sleep disturbances, apathy, ruminations, eating rituals) and/or a result of psychiatric comorbidities (major depressive disorder, anxiety disorder, obsessive compulsive disorder) (Keys et al. 1950). These symptoms tend to decrease during refeeding (Mattar et al. 2011; Pleplé et al. 2021; Godart et al. 2007). To date, study findings have agreed on clinical improvement in anxiety and depression symptoms after nutrition rehabilitation, and Pleplé et al. (2021) confirm a positive relationship between the course of eating disorder symptoms and that of anxiety‐depression symptoms during inpatient treatment of AN, even after adjustment on a vast array of possibly confounding factors (Mattar et al. 2012; Pleplé et al. 2021; Channon and DeSilva 1985; Coulon et al. 2009; Eckert et al. 1982; Kawai et al. 2008; Laessle et al. 1988; Meehan et al. 2006; Pollice et al. 1997; Brockmeyer et al. 2012; Sala et al. 2011).

Levels of anxiety, depression, and OC symptoms observed in states of undernourishment could be linked to multiple biological factors related to nutritional status, besides the anthropometric markers or albumin, which poorly reflect the kinetics and the scale of malnutrition, especially in AN (Mattar et al. 2012; Gauthier et al. 2014). Additionally, during weight loss and specifically in patients with an eating disorder (ED), significant changes in thyroid hormones have been observed, such as low T3 levels, normal or low T4 levels, and normal TSH levels, which tend to normalize during refeeding (de Zwaan et al. 2002; Onur et al. 2005). Additionally, Swenne et al. (2007) hypothesized that thyroid hormones (fT3, fT4, and TSH) could reflect the nutritional status of patients with AN (Swenne and Rosling 2010; Swenne et al. 2007) and potentially contribute to explaining the relationship between nutritional status and anxiety or depression symptoms (Swenne and Rosling 2010). They found that fT3, and to a lesser extent fT4 plasma levels, were indicative of nutritional status among patients with ED, whereby fT3 and fT4 levels are low and increase with refeeding, while TSH remains normal (Swenne et al. 2007). They also showed an association between low fT4 levels and depression symptoms, but no association with fT3 levels (Swenne and Rosling 2010).

On the other side, a link between depression and the thyroid hormone levels in the blood has been established and studied for decades, and could involve the cerebral metabolism, including serotonergic transmission (Escobar‐Morreale et al. 1995). Hypothyroidism, defined by low levels of thyroid hormones T3 and T4, and high TSH levels, is associated with an increased risk of depression (Fountoulakis et al. 2006), poor response to antidepressants, and an increased risk of relapse. However, the efficacy of antidepressant therapy when combined with the addition of treatment with tri‐iodothyronine (T3) is controversial. One meta‐analysis (Lorentzen et al. 2020) found inadequate proof to endorse the utilization of additional thyroid hormones in addressing unipolar depression that is resistant to treatment. Whereas (Nuñez et al. 2022) showed efficacy of thyroid hormones addition as an augmentation strategy to antidepressants. A possible reason for variations between our study and others in the thyroid literature might be attributed to differences in the study population, research design, variability in definitions of treatment‐resistant depression (TRD), and the therapeutic dosages employed in several studies encompassed in prior meta‐analyses.

To our knowledge, no study replicated the Swenne and Rosling work (2010), the latter entailing certain limitations related to the sample's average nutritional status and the depression diagnosis. Hence, given the insufficient studies on the topic, our objective was to study the link between depression, anxiety, and OC symptoms and the level of circulating thyroid hormones in a wide sample of undernourished patients with AN while considering the relative chronology of occurrence of comorbid diagnoses of depression and anxiety and other confounding factors described in the literature. Therefore, it can be theorized that in a large sample size of undernourished AN patients, thyroid hormones (fT3, fT4, and TSH) could mirror the nutritional status of the patients and possibly contribute to explaining the relationship between nutritional status and symptoms of depression, anxiety, and OC, regardless of its comorbidity.

## 2 Materials and Methods

### 2.1 Ethical Considerations

This study was part of a wider study on the assessment of hospitalization of anorexia nervosa known as EVHAN (n°EudraCT: 2007‐A01110‐53, registered on Clinicaltrials). The study protocol was approved by the Ethics Committee Ile‐de‐France III and the CNIL (Commission Nationale de l'Informatique et des Libertés). Written informed consent was obtained from each patient before inclusion in the study, in agreement with the Helsinki Declaration.

### 2.2 Clinical Sample

**FIGURE 1:**
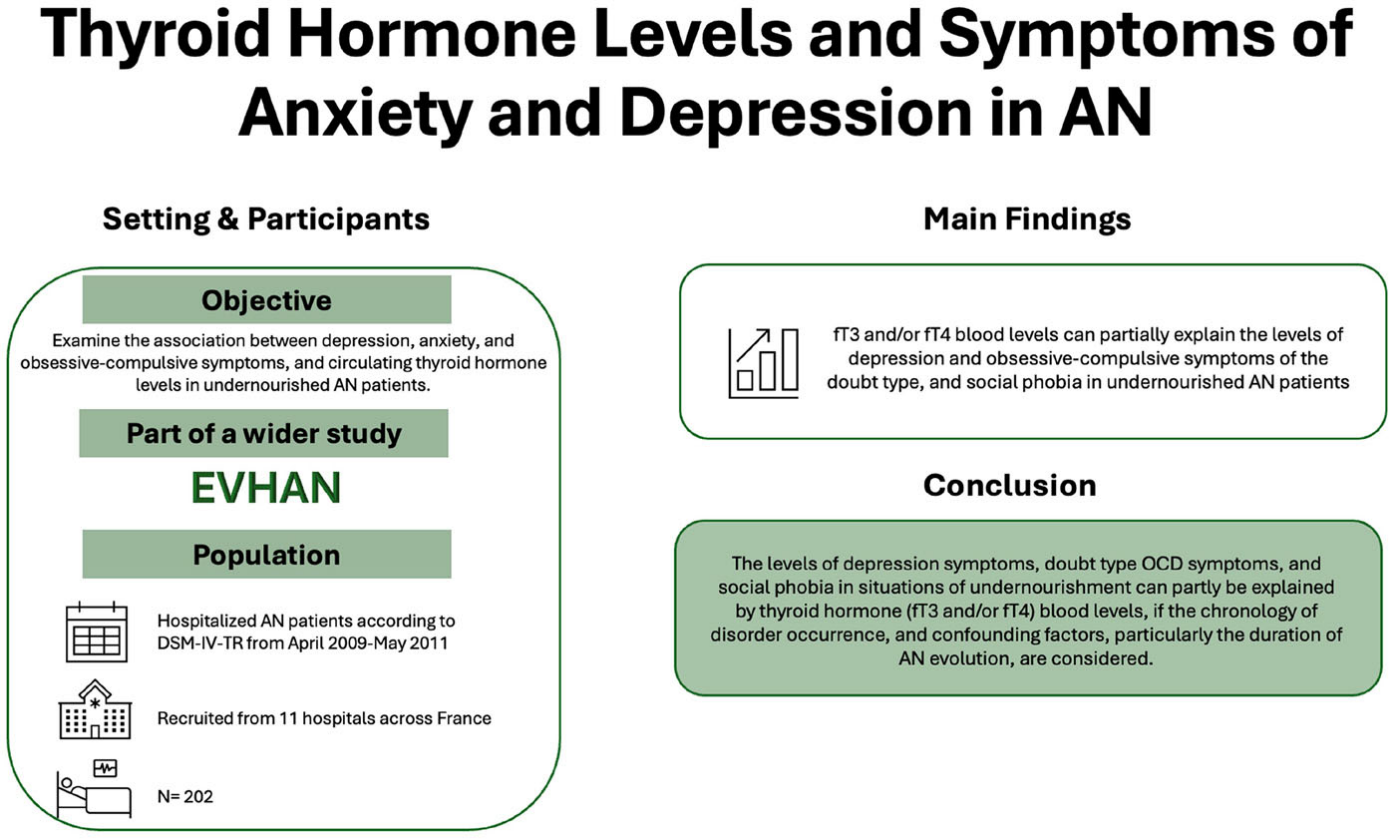
**Flow diagram**.

Patients hospitalized consecutively from April 2009 to May 2011 for AN according to the DSM‐IV‐TR criteria were included in the present study. The full EVHAN study methods have been described previously (Riquin et al. 2021). Patients were recruited from 11 hospitals across France. In total, 233 patients were included in the EVHAN study. At inclusion, 73% of the patients met all the DSM‐IV criteria, while the others presented a partial form of the disorder. 10 male patients, 11 patients under 13 years of age, and 10 patients from a center that did not carry out a full biological assessment, were excluded (Figure 1).

### 2.3 Clinical Assessment

#### 2.3.1 Diagnostic Assessment of AN

AN diagnosis was obtained using the Composite International Diagnostic Interview (CIDI 3.0) with BMI criteria (BMI < tenth percentile up to 17 years of age and BMI < 17.5 Kg/m^2^ from 17 years of age) and with the eating disorder examination questionnaire (EDE‐Q) for the AN sub‐type [restrictive (AN‐R) and bulimic with vomiting or purging (AN‐BP)]. The duration of the evolution of the disorder and the age at onset were collected, as well as the primary or secondary nature of amenorrhea.

A global assessment of the clinical state, psychiatric and medical histories, family relationships, and quality of life was done at the start and end of hospitalization. Lifetime comorbidities with major depressive disorder (MDD), anxiety disorder, and obsessive‐compulsive disorder (OCD) were assessed using the Short‐form CIDI (CIDI‐SF) (Kessler et al. 1998). Thirty‐eight out of the two hundred two patients could not be assessed. These 38 patients were compared to those assessed using the CIDI‐SF (*n* = 164) upon admission with no difference (*p*< 0.05), on all variables considered (depression, anxiety, social anxiety, OC behavior symptoms, body mass index (BMI), lean body mass (LBM), body fat percentage (BFP), age at admission, age at anorexia nervosa onset, and duration of evolution).

#### 2.3.2 Assessment of Nutritional Status

##### 2.3.2.1 Anthropometry and Body Composition

Weight was measured for each patient in the morning, nil‐by‐mouth and in underwear, and stature was measured upon admission. BMI was calculated (BMI = weight/height^2^) and expressed in kg/m^2^. The severity of malnutrition was estimated as the difference between reported BMI before illness and BMI upon admission to hospital.

Body composition was measured using bioelectric impedance analysis (BIA). It was assessed in the first two weeks of hospitalization (Kyle et al. 2004). Bioelectric impedance was measured using a bioelectrical analyzer (FORANA, Helios, Frankfurt, Germany) (Mattar et al. 2011). In this study, the fat mass index (FMI = FM/height^2^) and fat‐free mass index (FFMI = FFM/height^2^) were used rather than fat mass (FM) and fat‐free mass (FFM), as an adjustment on stature in a heterogeneous sample is essential to avoid biased comparisons.

#### 2.3.3 Assessment of Anxiety, Depression, Obsessive‐Compulsive, Social Phobia, and Eating Symptoms

Five questionnaires were used to assess the levels of depressive, anxiety, OC, social phobia, and eating symptoms.

##### 2.3.3.1 Beck's Depression Inventory (BDI)

This is a self‐administered questionnaire of 21 items assessing cognitive and emotional symptoms of depression at the time of administering the questionnaire (Beck and Beamesderfer 1974, Bourque and Beaudette 1982). The score ranges from 0 to 39.

##### 2.3.3.2 Hospital Anxiety and Depression Scale (HADS)

This is a self‐administered questionnaire of 14 items assessing the most frequent depression and anxiety symptoms. Intensity is also measured by considering the somatic aspects that can have an impact on the score. Only the anxiety dimension was considered in the results of this study (Lepine et al. 1985; Zigmond and Snaith 1983). The maximum total for anxiety is 21 points.

##### 2.3.3.3 Liebowicz's Social Anxiety Scale (LSAS)

This includes a clinical interview divided into two series of questions on fear and avoidance behaviors towards situations of social interaction and performance at the time of the interview. The two dimensions are strongly correlated, so only the fear dimension was considered in the results of this study (Yao et al. 1999). The score ranges from 55 (moderate social phobia) to 95 (very serious social phobia).

##### 2.3.3.4 Maudsley's Obsessive‐Compulsive Inventory (MOCI)

This is a self‐administered questionnaire assessing obsessive and compulsive behaviors via 30 items with a true/false format, divided into four sub‐scales: checking compulsions, compulsive washing/cleaning, slowness and repetition, and doubting/intrusive thoughts. The maximum score is 17 (Hantouche and Guelfi 1993; Hodgson and Rachman 1977).

##### 2.3.3.5 Eating Attitudes Test ‐26 (EAT‐26)

This is a self‐administered questionnaire assessing preoccupations and abnormal eating behaviors in an adult population. It comprises 26 items, divided into three subscales: dieting (including preoccupations with slimness), bulimia (and its associated symptoms, such as preoccupations about food), and oral control (example: self‐control regarding food). A score of 20 or over shows the presence of preoccupations regarding weight, silhouette, and feeding (Leichner et al. 1994; Garner et al. 1982).

#### 2.3.4 Assessment of Biological Data

Upon patient admission, fT3, fT4, and TSH thyroid hormone plasma levels were collected, as well as albumin and transthyretin levels. Thyroid hormone levels were measured in serum using a one‐step immunoassay, with antibodies marked using radioactive (RIA), enzymatic (EIA), or luminescent (LIA) markers (Sapin and Schlienger 2003). Values were adjusted to the reference norms of each center. Since assays had been performed by different laboratories, to homogenize the results, we divided each level of albumin and transthyretin (pre‐albumin) and of fT3, fT4, and TSH by the median value from each laboratory's reference interval.

#### 2.3.5 Other Parameters

Information on ongoing drug treatment was collected. The antidepressants were selective serotonin reuptake inhibitors, and the anxiolytics were benzodiazepines and antihistaminic drugs. No patient was receiving thyroid hormone replacement therapy.

### 2.4 Statistical Data Management

Statistics were conducted using Statistics Software Program (SPSS 23.0), with a nominal threshold of error (alpha risk) fixed at 5%. Results are reported as values, means, and standard deviations (± SD) for continuous variables.

The relationships between the different psychometric scores assessing the levels of depressive (BDI), anxiety (HADS and LSAS), and OCD (MOCI with its four sub‐dimensions: compulsive checking, washing and cleaning, slowness and repetition, and doubt and awareness) symptoms and the nutritional markers (BMI, FM, FFM, albumin and pre‐albumin levels, and thyroid hormones: fT3 and fT4) were assessed using Pearson's correlation analysis. Pearson's correlation was also applied to examine the associations between psychometric scores and potential quantitative confounding variables described in the literature (age at AN onset, age at admission, and duration of AN evolution) (Mattar et al. 2011) to assess their influence on psychological symptoms. Regarding the chronology of occurrence of the disorders in the case of comorbidities and possible qualitative confounding factors (AN sub‐type, antidepressants at admission), Student t‐tests were conducted. These statistical analyses, for exploratory purposes, were conducted without Bonferroni correction of the degree of statistical significance for multiple tests to identify all markers potentially associated with the criterion of interest (Rothman 1990). Given that the age at onset and age at admission were correlated (*r* = 0.65; *p* < 0.001), we only retained the age at admission; and as the duration of evolution and age at admission were correlated (*r* = 0.68 and *p* < 0.001), we decided to only retain the duration of evolution in the models.

All statistical tests were two‐tailed. Normality of distributions was visually inspected and confirmed using Shapiro‐Wilk tests when necessary.

#### 2.4.1 Multivariate Analyses

Multiple linear regressions were performed to test the links between the psychometric scores for depressive (BDI), anxiety (HADS, LSAS fear), and OCD (MOCI: four sub‐dimensions: compulsive checking, washing and cleaning, slowness and repetition, and doubt and awareness) symptoms and the variable of interest, i.e., the levels of thyroid hormones (fT3, fT4). Furthermore, the chronology of occurrence of the disorders was systematically introduced into these models (existence of a disorder preceding AN), and the possible confounding factors identified from the literature and significantly linked to the dependent variable in univariate analyses (*p* < 0.05). Specifically, age at admission, duration of AN, and antidepressant use were retained based on significant univariate associations and theoretical justification. We ran a model for each thyroid hormone (fT3 and fT4). The selection of variables to be included in the models was first performed on the basis of clinical arguments and results from association analyses. No technique for model selection (ascending or descending) was used a‐posteriori. The assumptions of linear regression were checked and met for each model.

## 3 Results

### 3.1 Clinical Characteristics

At admission, half of the participants (*n* = 101) had AN of the restrictive type (AN‐R), and the other half had AN of the binge‐eating/purging type (AN‐BP). Patients’ mean age at admission was 20.80 ± 6.58 years, with a mean age at illness onset of 16.37 ± 4.27 years. The mean duration of evolution of the disorders was 4.04 ± 4.16 years. Participants’ nutrition status characteristics at admission are presented in Table 1.

**TABLE 1:** Patients’ nutritional characteristics at admission

	*N*	Mean ± SD
**BMI (kg/m^2^)**	202	14.27 ± 1.51
**FFMI (kg/m^2^)**	187	12.48 ± 0.82
**FMI (kg/m^2^)**	179	1.92 ± 1.15
**fT3 levels** ^*^ **(ng/dL)** *(normal ranges 80–220 ng/dL)*	179	0.73 ± 0.21
**fT4 levels** ^*^ **(µg/dL)** *(normal ranges 5.0–12.0 µg/dL)*	189	0.81 ± 0.14
**TSH levels** ^*^ **(mU/L)** *(normal ranges 0.4–4.0 mU/L)*	195	0.81 ± 0.42
**Albumin levels** ^*^ **(g/dL)** *(normal ranges 3.5–5.5 g/dL)*	183	1.04 ± 0.15
**Transthyretin levels** ^*^ **(mg/dL)** *(normal ranges 18–45 mg/dL)*	163	0.86 ± 0.19

**Abbreviations**: BMI, body mass index; FFMI, fat‐free mass index; FMI, fat mass index.

* Ratio between each patient's level and the central value from each laboratory's reference interval.

### 3.2 Comorbidities and Relative Chronology

More than half of the subjects, 56.1% (*n* = 92/164), presented a major depressive episode (MDE) in the course of their life. Among them, 27.8% (*n* = 45/162), had major depressive disorder (MDD) before AN, and for the others, it was after or concomitant. Lifetime generalized anxiety disorder (GAD) was present for 32.3% (*n* = 53/164) of the subjects. For 17.6% (*n* = 28/159) of the subjects, its occurrence was before AN onset. Furthermore, 25.6% (*n* = 42/164) of the sample presented lifetime OCD. For 18.5% (*n* = 30/162), it was before the onset of AN. Finally, 30.5% (*n* = 50/162) of the sample presented a lifetime comorbidity of the social phobia type. For 21.2% (*n* = 35/165) of the subjects, it occurred before AN onset.

### 3.3 Scores on Psychometric Scales

The mean score for the BDI depression scale (26.78 ± 11.93) corresponds to a severe level of depression. As for the HADS anxiety scale (anxiety dimension) (12.44 ± 4.38), the mean score corresponds to anxiety symptoms of average intensity. The mean score for the MOCI obsessions and compulsions scale (11.51 ± 5.29) corresponds to the presence of moderate OCD. The mean score for EAT‐26 (35.66 ± 16.51) indicates the presence of preoccupations with regard to weight, silhouette, and feeding.

### 3.4 Relationship Between Psychological Symptoms and Markers of the Nutritional State

We did not find any link between depressive, anxiety, or OCD symptoms and the nutritional parameters (BMI, FFMI, FMI, and transthyretin (TTR))or the thyroid hormones (fT3 and fT4), with the only exception being albumin levels. The lower the albumin level, the higher the level of social phobia (*r* = ‐0.21; *p* = 0.006).

### 3.5 Relationship Between Psychological Symptoms and Possible Confounding Factors

The longer the duration of AN evolution, the higher the levels of depressive symptoms (BDI score: *r* = 0.19; *p* = 0.008; *n* = 194), anxiety symptoms (HADS score: *r* = 0.25; *p* = 0.0001; *n* = 200), and social phobia symptoms (LSAS score for fear sub‐scale: *r* = 0.21; *p* = 0.004; *n* = 197). As for the OCD symptoms, the results were not significant (MOCI score: *r* = 0.11; *p* = 0.110; *n* = 197).

AN‐BP patients had on average a higher level of depressive symptoms than AN‐R patients (respective BDI scores: 28.76 ± 11.50 versus 24.75 ± 12.09; *p* = 0.018). On the other hand, the mean scores did not differ for the HADS, MOCI, and LSAS scales.

There was a significant link between the chronology of occurrence of a major depressive episode (MDE) and the depression score measured on the BDI (34.07 ± 11.30, *p* < 0.0001 versus 24.02 ± 11.22), between the chronology of occurrence of OCD and the MOCI score (14.62 ± 4.65 versus 10.80 ± 5.30, *p* < 0.0001), and between the chronology for social phobia disorder and the LSAS score (42 ± 14.05 versus 29.18 ± 15.42, *p* < 0.0001). However, there was no link between the chronology of occurrence of generalized anxiety disorder and the HADS score (*p* = 1.121).

The existence of antidepressant treatment was associated with a significantly higher level of depressive, anxiety, and OCD symptoms for the scales used: BDI (32.38 ± 11.20 versus 23.99 ± 11.33; *p* < 0.0001), HADS (13.97 ± 3.81 versus 11.68 ± 4.46; *p* < 0.0001), MOCI (12.68 ± 5.29 versus 10.93 ± 5.22; *p* < 0.001), and LSAS (38.56 ± 15.77 versus 28.11 ± 14.55; *p*< 0.0001), respectively.

### 3.6 Multivariate Models

Separate multivariate analyses were performed to ascertain whether blood levels of fT3 (Table 2) and fT4 (Table 3) thyroid hormones could contribute to explaining the psychometric scores once confounding factors previously identified in univariate analysis for each scale and the chronology of disorder occurrence were considered.

**TABLE 2:** Multivariate analyses fT3

Dependant variables	Explanatory variables	*r^2^*; *f*; *p*	*ß*	*p*
BDI score	**fT3 levels**	**0.195**	**−0.16**	**0.039**
	Anorexia sub‐type	**f = 8.366**	−0.08	0.288
	Duration of AN evolution	***p* < 0.0001**	0.19	0.013
	**Chronology of disorder occurrence**		**0.35**	**< 0.0001**
HADS anxiety score	fT3 levels	**0.079**	−0.12	0.146
	**Duration of AN evolution**	***f* = 4.002**	**0.24**	**0.005**
	Chronology of disorder occurrence	***p* = 0.009**	0.09	0.277
MOCI score “checking”	fT3 levels	0.034	−0.03	0.705
		*f* = 2.469		
	Chronology of disorder occurrence	*p* = 0.088	0.18	0.03
MOCI score “cleaning”	fT3 levels	**0.054**	−0.06	0.475
		***f* = 4.046**		
	Chronology of disorder occurrence	***p* = 0.020**	**0.23**	**0.007**
MOCI score “slowness”	fT3 levels	0.014	−0.1	0.223
		*f* = 1.035		
	Chronology of disorder occurrence	*p* = 0.358	0.06	0.444
MOCI score “doubting”	**fT3 levels**	**0.064**	**−0.19**	**0.019**
		***f* = 4.816**		
	**Chronology of disorder occurrence**	***p* = 0.009**	**0.16**	**0.044**
LSAS fear sub‐scale score Social phobia dimension	**fT3 levels**	**0.239**	**−0.16**	**0.043**
	**Duration of AN evolution**	***f* = 10.309**	**0.25**	**0.001**
	**Albumin levels**	***p* < 0.0001**	**−0.19**	**0.017**
	**Chronology of disorder occurrence**		**0.27**	**0.001**
LSAS fear sub‐scale score	fT3 levels	**0.187**	0.01	0.928
Performance phobia dimension	**Duration of AN evolution**	***f* = 7.419**	**0.2**	**0.014**
	Albumin levels	***p* < 0.0001**	−0.14	0.081
	**Chronology of disorder occurrence**		**0.33**	**< 0.001**

**TABLE 3:** Multivariate analyses fT4

Dependant variables	Explanatory variables	*r^2^*; *f*; *p*	*ß*	*p*
BDI score	Ft4 levels	0.186	−0.12	0.128
	Anorexia sub‐type	***f* = 8.356**	−0.14	0.069
	**Duration of AN evolution**	***p* < 0.0001**	**0.17**	**0.029**
	**Chronology of disorder occurrence**		**0.34**	**< 0.0001**
HADS anxiety score	fT4 levels	**0.059**	0.06	0.429
	**Duration of AN evolution**	***f* = 3.070**	**0.2**	**0.014**
	Chronology of disorder occurrence	***p* = 0.030**	0.08	0.316
MOCI score “checking”	fT4 levels	**0.043**	−0.07	0.379
		***f* = 3.410**		
	**Chronology of disorder occurrence**	***p* = 0.036**	**0.19**	**0.019**
MOCI score “cleaning”	fT4 levels	**0.063;**	−0.07	0.363
		***f* = 5.051**		
	**Chronology of disorder occurrence**	***p* = 0.008**	**0.24**	**0.004**
MOCI score “slowness”	fT4 levels	0.007	−0.01	0.919
		*f* = 0.508		
	Chronology of disorder occurrence	*p* = 0.603	0.08	0.323
MOCI score “doubting”	**fT4 levels**	**0.062;**	**−0.18**	**0.022**
		***f* = 4.960**		
	**Chronology of disorder occurrence**	***p* = 0.008**	**0.15**	**0.053**
LSAS fear sub‐scale score	fT4 levels	**0.192**	−0.05	0.505
Social fear dimension	**Duration of AN evolution**	***f* = 8.219**	**0.22**	**0.004**
	**Albumin levels**	***p* < 0.0001**	**−0.18**	**0.03**
	**Chronology of disorder occurrence**		**0.29**	**< 0.001**
LSAS fear sub‐scale score	fT4 levels	**0.187**	−0.03	0.682
Performance phobia dimension	**Duration of AN evolution**	***f* = 7.800**	**0.21**	**0.007**
	Albumin levels	***p* < 0.0001**	−0.11	0.171
	**Chronology of disorder occurrence**		**0.34**	**< 0.001**

#### 3.6.1 Depression Symptoms (Score on the BDI Scale)

The level of depressive symptoms upon admission (score on the BDI scale) is significantly explained by levels of fT3 hormones, the duration of AN evolution, and the chronology of disorder occurrence when AN sub‐type is considered. The model explained 19.5% of the variance. The level of depression symptoms was proportionally higher when the level of fT3 was low, when the duration of AN evolution was long, and when patients presented a major depressive episode (MDE) prior to AN. The level of depression symptoms is not explained by fT4 levels.

#### 3.6.2 Anxiety Symptoms (HADS Anxiety Score)

The level of anxiety symptoms upon admission measured on the HADS score is not explained by either the fT3 or the fT4 thyroid hormone level. But it was explained in both models by the duration of AN evolution.

#### 3.6.3 OCD Symptoms (MOCI Score)

Only the fourth sub‐dimension (doubting/intrusive thoughts) in the MOCI score was linked to levels of thyroid hormones. The level of doubt/intrusive thought was proportionally higher when fT3 and fT4 levels were low and when AN occurred prior to OCD (for the model with fT3 and for the model with fT4). The model with fT3 levels explained 6.4% of the variance, and the model with fT4 levels explained 6.2% of the variance.

#### 3.6.4 Symptoms of Social Phobia (LSAS Score)

Only the social fear sub‐scale in the LSAS score was correlated with fT3 levels but not with the fT4 levels. The lower the fT3 levels, the longer the duration of evolution, the lower the albumin levels, and when AN occurred prior to the social phobia, the higher the level of social phobia symptoms. The model explained 23.9% of the variance.

#### 3.6.5 Other Analyses

The analyses were conducted by adding to the initial models the existence of an antidepressant treatment (AD), which did not change the results in any way, except that ADs were all significantly associated with a high level of anxiety, depression, and OCD symptoms upon admission.

## 4 Discussion

We found a link between the degree of adaptation to prolonged semi‐fasting expressed by the blood levels of thyroid hormones (fT3) and the level of depression symptoms (BDI score), social anxiety symptoms (fear sub‐scale score in the LSAS score), and OCD symptoms (sub‐score doubt and awareness on the MOCI), taking the chronology of occurrence of the comorbidities studied into account (respectively MDE, GAD, social phobia, and OCD) and the other confounding variables identified (including, for almost all symptoms, duration of AN evolution and albumin for social phobia symptoms). This link was not evidenced in the univariate analyses, whether with thyroid hormones or with other markers of the nutritional state, unlike previous studies (Mattar et al. 2011; Mattar et al. 2012; Mattar et al. 2012).

With regard to the level of depression symptoms, they were proportionally higher when undernourishment was high or more severe (low fT3 levels), when the duration of AN evolution was long (as classically described in cases of chronic illness (Steinhausen 2002)), and when the patients presented an MDE prior to AN. Concerning thyroid hormones, our results concur in part with Brambilla et al.'s work, in which they found that in a sample of 65 AN‐R patients, the depression sub‐scale SCL‐90R was negatively correlated to fT3 and fT4 blood levels adjusted on BMI (Brambilla et al. 2006). Swenne's team also examined this issue, considering a sample of 239 patients with various eating disorders, some of whom were not undernourished (Swenne and Rosling 2010). They showed a link between low fT4 levels and the existence of a current depressive episode (MADRS score > 18 or a clinical diagnosis of depression) but no link to fT3 levels. This could be explained, in addition to the difference in sample size, by considering the variables in the regression model. Indeed, they used BMI, weight loss, the weight loss per day, and the duration of illness evolution, but not a history of comorbidities or their chronology, nor any medication.

Similarly, the level of OCD symptoms measured on the doubt/awareness MOCI sub‐dimension was proportionally higher when fT3 and fT4 levels were low and when the patients presented an OCD prior to AN. To our knowledge, there are no data on OCD symptoms and thyroid hormones. Our study found a link between the level of social phobia and the nutritional state, which has not been demonstrated to date: the level of symptoms of the social phobia type measured on the LSAS fear sub‐scale score was proportionally higher when the fT3 levels were low (indicating high levels of undernourishment), when the duration of evolution was long, when albumin levels were low, and when patients presented a disorder of the social phobia type prior to AN. As suggested by Brambilla and Swenne's teams (Swenne and Rosling 2010; Brambilla et al. 2006), it could be argued that eating disorder symptoms cause undernourishment, which as a consequence leads to a decrease in circulating thyroid hormone levels, which in turn leads to a decrease in the bio‐availability of thyroid hormones to the brain and an action on cerebral serotonin, thus leading to an increase in anxiety and depression symptoms.

Moreover, it has been established in the literature that thyroid hormones influence the cerebral metabolism and monoaminergic transmission in particular (Whybrow and Prange 1981). Given fT3's role in boosting glucose utilization and ATP production in brain cells, thyroid hormones support the brain's high energy demands and neural activity (Whybrow and Prange 1981). Many studies have demonstrated an existing clear link between serotoninergic transmission and depression symptomatology, specifically in patients with hypothyroidism who present reduced levels of cerebral serotonin (5‐HT), which are reversible following treatment with thyroid hormones (Blier and de Montigny 1994; Nuguru et al. 2022). Thyroid hormones have therefore been linked to regulating the expression of enzymes involved in neurotransmitter synthesis and reuptake of transporters for serotonin and dopamine (Whybrow and Prange 1981; Blier and de Montigny 1994; Nuguru et al. 2022). Studies on animals (rats) have been able to show that thyroid hormones increase serotoninergic neurotransmission by reducing the sensitivity of 5‐HT1A auto‐receptors in the raphe nuclei and by increasing the sensitivity of 5‐HT2 receptors (Bauer et al. 2002). In adult rats, hypothyroidism, a low level of plasmatic thyroid hormones, has an impact on the brain and synaptic plasticity, inducing behavior of the depressive type and changes in learning and memory processes, including the potentiation of memory regarding fear (Fernández‐Lamo et al. 2009). As hypothyroidism is being linked to low serotonin and dopamine activity, low circulating thyroid hormones are contributing to depression, and T3 is being added to antidepressant treatments for patients with depression (Blier and de Montigny 1994; Nuguru et al. 2022; Bauer et al. 2002). Hypothyroidism induces reversible depressive symptoms, which are correlated with changes in neurogenesis; this suggests that mood symptoms in adults linked to hypothyroidism could be partly due to an impact on neurogenesis (Bauer et al. 2002). Drawing on this consensus from a therapeutic point of view, the international recommendations (Yager et al. 2006; Recommandations professionnelles 2007; Excellence NIfHaC 2020) advise refeeding in case of an MDE in an undernourished AN patient before considering any antidepressant treatment (Leble et al., 2017).

As part of the endocrine dysregulation seen in AN patients and aside from dysfunction of the hypothalamic‐pituitary axis, AN can be associated with alterations in adipokines and appetite‐regulating hormones (Schorr and Miller 2017). This could be another hypothesis suggesting the potential link between decreased deiodination of fT3 and fT4 with depressive behaviors in AN patients could be low levels of leptin (Schorr and Miller 2017; Heinen et al. 2023). Leptin, known for its regulatory role in body weight and metabolism, has recently emerged as a factor influencing mood and cognition by modulating synaptic changes in the brain, similar to antidepressant effects (Schorr and Miller 2017). As leptin levels tend to be low in AN patients due to the significant loss of body fat associated with the disorder, depressive behaviors might arise.

However, our result has enabled us to confirm that patients with a pre‐morbid medical history of depression have higher levels of depression symptoms, independently from their nutritional state (assessed by fT3 levels) and the duration of the disorder. The level of depression symptoms at admission measured on the BDI scale in very undernourished patients in our study was linked to low fT3 levels, which could reflect cerebral levels, with a consequent disruption of cerebral serotoninergic transmission and an increase in the level of depression symptoms.

It is noteworthy to also mention that the dysregulation of the hypothalamic‐pituitary‐thyroid axis in AN patients mirrors patterns seen in euthyroid sick syndrome (Gibson 2023). Euthyroid sick syndrome, also known as nonthyroidal illness syndrome, encompasses transient alterations in thyroid function tests observed primarily in critically ill patients, often in the context of severe illness, calorie deprivation, or major surgeries (Ganesan and Wadud 2023). In both AN and euthyroid sick syndrome, low total and free fT3 levels are often observed, while fT4 and TSH levels tend to be within the low to normal range (Gibson 2023, Ganesan and Wadud 2023). Notably, fT4 is converted to the inactive reverse fT3 instead of the active form, serving to conserve energy. In such cases, thyroid supplementation is discouraged in AN, as these changes are physiologic and resolve with weight restoration (Gibson 2023). As previously mentioned, there is a potential link between decreased fT3 levels and depressive symptoms in acutely underweight AN patients, suggesting ongoing associations between thyroid hormone conversation ratios and psychopathology even post‐weight restoration (Wronski et al. 2022). Such findings inform clinical practices, including thyroid monitoring and experimental low‐dose thyroid hormone supplementation in severe cases of AN with depression and significant thyroid hormone changes (Wronski et al. 2022).

To summarize, anxiety and depression symptomatology in AN during an undernourishment phase appears as a sort of chimera, gathering symptoms linked to AN symptoms, undernourishment consequences of food restriction, genuine comorbid anxiety or depressive disorders, and finally, links to chronicity as in all chronic illnesses (Chronic Illness and Mental Health: Recognizing and Treating Depression 2021). According to some studies (Godart et al. 2007), certain patients (between 20 and 25%) present depression or OCD or social phobia symptoms occurring prior to AN. In addition, in the phase of undernourishment as a result of AN, all patients experience more severe depression, OCD, and social phobia symptoms, which are linked to undernourishment via a decrease in fT3 and/or fT4 blood levels (Swenne and Rosling 2010) and/or other biological factors such as tryptophan or serotonin (Gauthier et al. 2014). AN is a disorder that evolves over several years, and its chronicity aggravates depressive characteristics and social and affective isolation, particularly via a decrease in social interactions linked to symptoms of social phobia (Steinhausen 2002; Schmidt 2005). Among patients with a disorder prior to AN, symptoms linked to the evolution of AN are added to pre‐existing ones, and these patients have a particularly severe prognosis (Steinhausen 2002; Carrot et al. 2017).

These issues raise the question of the optimal moment for treating anxiety or depressive disorders in AN, particularly for whom antidepressant (AD) treatment could be prescribed for these patients in order to improve prognosis. AD treatments have shown their inefficacy in AN in general (Mischoulon et al. 2011; Guarda 2008). We hypothesize that this result is linked to the difficulty of diagnosing anxiety or depressive disorders in AN as a high level of symptoms could be due to malnutrition only and not a comorbidity, and that a targeted indication of AD for patient profiles with depression, OCD, or social phobia comorbidities preceding AN could be a therapeutic option (Leblé et al. 2017). Hence, early care provision for these disorders, even for undernourished patients, could enable improvement in their prognosis, since depression, OCD, and social phobia interfere with refeeding and overall eating disorder treatment. However, this hypothesis needs to be explored, as it has been shown that low fT3 levels could be an obstacle to AD efficacy (Bauer et al. 2002). However, the introduction of ADs during refeeding, if possible not immediately, could occur earlier in this case, as fT3 levels normalize rapidly after refeeding, and hence it is no longer necessary to wait for weight normalization (Swenne et al. 2007).

### 4.1 Strengths and Limitations

This study presents certain limitations that could interfere with the results or limit their generalization. We considered as “previous to AN onset” any disorder for which age at onset was at least a year before AN onset. This could imply a possible patient recall bias, leading us to underestimate disorders that started before AN but at the same age. Additionally, this study included only hospitalized AN patients, which may not be representative of all AN patients.

Furthermore, the absence of a control group of age‐matched healthy individuals or patients with other eating disorders restricts our ability to determine whether the observed associations are specific to anorexia nervosa or reflect broader effects of malnutrition. Second, due to its cross‐sectional design, the study cannot establish causal relationships between thyroid hormone levels and psychiatric symptoms. However, the study's naturalistic, prospective design, based on routine clinical assessments at the point of hospitalization, reflects real‐world conditions. Future research employing longitudinal designs and including comparative groups would be valuable to explore these associations over time and across populations.

We studied plasma levels for free thyroid hormones (fT3 and fT4), and it is these free hormones that are biologically active, acting as very good mirror images of T3 and T4 plasma levels, but not of cerebral levels. Our results mainly concerned T3, which does not cross the blood‐brain barrier. Nevertheless, several arguments favor it as a good indirect marker of the state of cerebral bio‐availability of thyroid hormones. In AN, the thyroid decreases production of T3 and T4, which is an adaptive physiological mechanism to undernourishment, enabling energy metabolism and mood disorders to be reduced. However, there is an obstacle to the peripheral conversion of T4 into T3 (20% of T3 comes from the thyroid, and 80% comes from conversion). Furthermore, only T4 can cross the blood‐brain barrier, with the help of transthyretin. However, it has been demonstrated that T4 blood levels, though normal, are low and that transthyretin blood levels, on the other hand, are reduced in states of undernourishment. These two mechanisms could contribute, as is the case with depression, to a decrease in T4 bio‐availability, and therefore a decrease in T3 cerebral bio‐availability. This stems from T4 deiodination through iodothyronine deiodinases type 2. From a methodological viewpoint, a centralization of the biological analyses in this study was not possible because of costs and the laborious nature of the procedure.

One of the strengths of this study in comparison with other studies is that we considered a large, homogenous sample of severely undernourished AN patients (BMI at admission as low as 14.3). Furthermore, we widened the range of symptoms under investigation, as we examined, in addition to depression, anxiety, and obsessive symptoms. Finally, alongside symptom levels, we also took the relative chronology of occurrence with AN of depression and anxiety comorbidities (MDE, GAD, social phobia, and OCD) and the existence of antidepressant treatments into account. The consideration of a medical history of anxiety or depression disorder prior to AN and the duration of the disorder enabled true anxiety‐depression symptoms to be differentiated, i.e., those that were a comorbid disorder and depression symptoms linked to undernourishment.

## 5 Conclusion

The levels of depression symptoms, doubt‐type OCD symptoms, and social phobia in situations of undernourishment can partly be explained by thyroid hormone (fT3 and/or fT4) blood levels if the chronology of disorder occurrence and confounding factors, particularly the duration of AN evolution, are considered. The existence of MDE, OCD, or social phobia previous to AN aggravates its prognosis, and this is therefore associated with more severe symptoms of depression or OCD at admission. In clinical practice, a high level of these symptoms should trigger an investigation into any associated comorbidities and raise issues on a case‐by‐case basis for the early implementation of specific treatment, which should be independent from the process of refeeding. Future studies are needed to verify if the improvement in fT3 levels is correlated with the reduction of anxiety and depressive symptoms. The persistence of high depressive and anxiety symptoms despite normalization of fT3 and/or a pre‐existing comorbidity with AN could argue for the implementation of antidepressant treatment. The effectiveness of such a strategy should be evaluated in both inpatient and outpatient AN populations.

## 6 Public Significance Statement

Our novel approach illuminates the connection between undernourished AN patients and symptoms of depression, anxiety, and OCD with thyroid hormone levels. Taking into consideration the chronology of disorder occurrence and confounding factors, especially the duration of AN evolution, identifying pre‐existing mental health concerns previous to AN is critical and can enhance treatment outcomes. This emphasizes the necessity for thorough assessment and personalized interventions, potentially leading to more effective management strategies.

## Author Contributions

LM contributed to the conceptualization, methodology, and writing of the original draft. ALD collected data and analyzed the first draft. SBL was involved in formal analysis, and critical revision of the manuscript. MD contributed to investigation and data collection. SI supported methodology and visualization efforts. CL was responsible for formal analysis, software, and visualization. The EVHAN group participated in investigation and provision of resources. MH contributed to supervision, validation, and manuscript review. NG provided oversight, conceptual guidance, funding acquisition, and contributed to the final review and editing of the manuscript. LM is the corresponding author.

## Ethics Statement

This study was part of a wider study on the assessment of hospitalization of anorexia nervosa known as EVHAN, (n°EudraCT: 2007‐A01110‐53, registered on Clinical trials). This study protocol was approved by the Ethics Committee Ile‐de‐France III and the CNIL (Commission Nationale de l'Informatique et des Libertés).

## Consent

Written informed consent was obtained from each patient before inclusion in the study, in agreement with the Helsinki declaration.

## Conflicts of Interest

The authors declare no conflicts of interest.

## Peer Review

The peer review history for this article is available at https://publons.com/publon/10.1002/brb3.70685

## Data Availability

Data is available upon request.
